# Development of screening methods for functional characterization of UGTs from *Stevia rebaudiana*

**DOI:** 10.1038/s41598-020-71746-9

**Published:** 2020-09-15

**Authors:** Eva Petit, Monique Berger, Laurent Camborde, Veronica Vallejo, Jean Daydé, Alban Jacques

**Affiliations:** 1 Equipe Physiologie, Pathologie et Génétique Végétales (PPGV), INP-PURPAN, Université de Toulouse, 75 voie du TOEC, BP 57611, 31076 Toulouse Cedex 03, France; 2 Laboratoire de Recherche en Sciences Végétales (LRSV), CNRS, Université Paul Sabatier (UPS), Toulouse, France; 3 PepsiCo, 700 Anderson Hill Rd., Purchase, NY 10577 USA

**Keywords:** Biochemistry, Biological techniques, Biotechnology, Plant sciences

## Abstract

Glycosylation is a key modification that contributes to determine bioactivity and bioavailability of plant natural products, including that of terpenoids and steviol glycosides (SVglys). It is mediated by uridine-diphosphate glycosyltransferases (UGTs), that achieve their activity by transferring sugars on small molecules. Thus, the diversity of SVglys is due to the number, the position and the nature of glycosylations on the hydroxyl groups in C-13 and C-19 of steviol. Despite the intense sweetener property of SVglys and the numerous studies conducted, the SVglys biosynthetic pathway remains largely unknown. More than 60 SVglys and 68 putative UGTs have been identified in *Stevia rebaudiana*. This study aims to provide methods to characterize UGTs putatively involved in SVglys biosynthesis. After agroinfiltration-based transient gene expression in *Nicotiana benthamiana*, functionality of the recombinant UGT can be tested simply and directly in plants expressing it or from a crude extract. The combined use of binary vectors from pGWBs series to produce expression vectors containing the stevia's UGT, enables functionality testing with many substrates as well as other applications for further analysis, including subcellular localization.

## Introduction

Steviol glycosides (SVglys) are *ent*-kaurenoic diterpenoids produced and accumulated in *Stevia rebaudiana* leaves up to 25% of the dry matter^1^. Biosynthesis of SVglys starts with the formation of geranyl-geranyl diphosphate (GGDP) in the chloroplast from the methyl-erythritol-4-phosphate (MEP) pathway^2–4^. GGDP then undergoes two cyclization steps, catalyzed by terpene cyclases to give *ent*-kaurene^5^. *Ent*-kaurene is subsequently oxidized into kaurenoic acid (KA) by kaurene oxidase (KO) in the endoplasmic reticulum (ER). Kaurenoic acid is the last step of a biosynthetic pathway shared by gibberellins and SVglys. Oxidation of KA leads to gibberellins, while its hydroxylation produces steviol that can be glycosylated on the two hydroxyl groups of its C-13 and C-19 to give SVglys. Glycosylation is catalyzed by UDP (uridine diphosphate)-glycosyltransferases (UGTs). To form steviol glycosides, UGTs transfer an activated sugar of a sugar-donor substrate on hydroxyl groups of an acceptor substrate. Glycosylation is a key and widespread modification of secondary metabolites, playing a pivotal role in the huge diversity of plant natural products^6^. Thus, more than 60 SVglys have been identified in *S. rebaudiana* leaves to date^1^. Main SVglys are stevioside (ST), rebaudioside A, C, D, E, F (Reb A-F), dulcoside A (Dulc A) and steviolbioside. In *S. rebaudiana*, a wide phenotypic variability in SVglys profiles can be observed^7–9^. For example, some genotypes synthetize very high proportions of Reb A and/or Reb C, whereas others accumulate preferentially their corresponding substrates ST and Dulc A, respectively^10,11^. These differences could be due to variability of the enzyme responsible for Reb A and Reb C biosynthesis, UGT76G1^10,11^.

The first part of the SVglys biosynthesis leading to steviol formation is well known and characterized^12,13^. The latter part, leading to SVglys from the steviol aglycone remains largely unknown as *S. rebaudiana* UGTs are poorly characterized, preventing a better understanding of the SVglys pathway. Nevertheless, several UGT genes of *S. rebaudiana* are available. For example, 12 candidates have previously been identified^14^. In a more recent study, Li et al. isolated 144 sequences suspected to be UGTs by re-analyzing the public transcript data of *S. rebaudiana* from NCBI (SRR1576548), among them 68 were unambiguously identified as UGT genes^15^.

When a UGT gene is available, one the most common methods to identify its functionality is in vitro expression in a heterologous system^16^. Substrate specificity of plant UGTs involves recognition of a sugar acceptor as well as a UDP-sugar donor^17^. In most instances, UGTs show a strong specificity for the UDP-sugar; even those exhibiting activities toward several sugar donors, often have a better affinity for only one of them^17^. UGTs show broad differences in their individual number of possible acceptors. Some UGTs are highly specific and only glycosylate few acceptors. For example, UGT73A14 of *Gentiana triflora* specifically glucosylates the 3′-hydroxy group of delphinidin-type anthocyanins having glucose at 3 and 5 positions^18^. Many other UGTs are regiospecific, thus they can glycosylate a large array of compounds, often part of the same metabolic family. Possible acceptor substrates tested in vitro can also be different. This is the case of UGT76G1 of *S. rebaudiana*, which glucosylates alcohols, substituted monophenols, polyphenols and glycosides^19^. Identification of an enzyme for a single glycosylation step using in vitro tests is limited by the panel of substrates used. For example, UGT74G1 and UGT73E1 appear to be steviol C-4 glucosyltransferases giving the 19-*O*-glucoside in *S. rebaudiana*^14,15^. However, they were also found to glucosylate the same site to produce rubusoside from steviolmonoside. Additionally, these tests may disregard the substrate synergy present in plant or the affinity of enzymes for these different substrates. These ambiguities underpin the need to test the specificity of plant UGTs in vivo, to better understand the complex or uncommon metabolic pathways as well as the plasticity of metabolism in response to biotic and abiotic stresses.

A method to reveal the functionality of a plant UGT which takes into account their polyspecific nature, is the creation of mutants of which the phenotype is compared to the control plants. Two different methods are available to create genetically transformed plants: stable or transient transformations. Stable transformation consists in integrating a foreign DNA sequence in the genome of a plant of interest in order to modulate plant traits such as stress tolerance, disease resistance or metabolite production. In the opposite, transient transformation is a process leading to the introduction of a nucleic sequence with temporary expression and tissue specific localization. Transient transformation has several advantages compared to stable one, as it does not need to create stable lines.

This study deals with two simple and original methods to characterize functionality of stevia’s UGTs after transient expression in *Nicotiana benthamiana* with the caveat that this system is not intended to characterize enzyme kinetics. These methods (1) avoid the purification step generated by recombinant protein expression in micro-organisms or plants, and allow to (2) quickly screen the functionality of an enzyme with individual substrates, (3) test a mix of proven acceptors, which might give clues on the substrate affinity of the enzyme in vivo.

## Results

### Recombinant proteins are expressed in *Nicotiana benthamiana*

**Figure 1:**
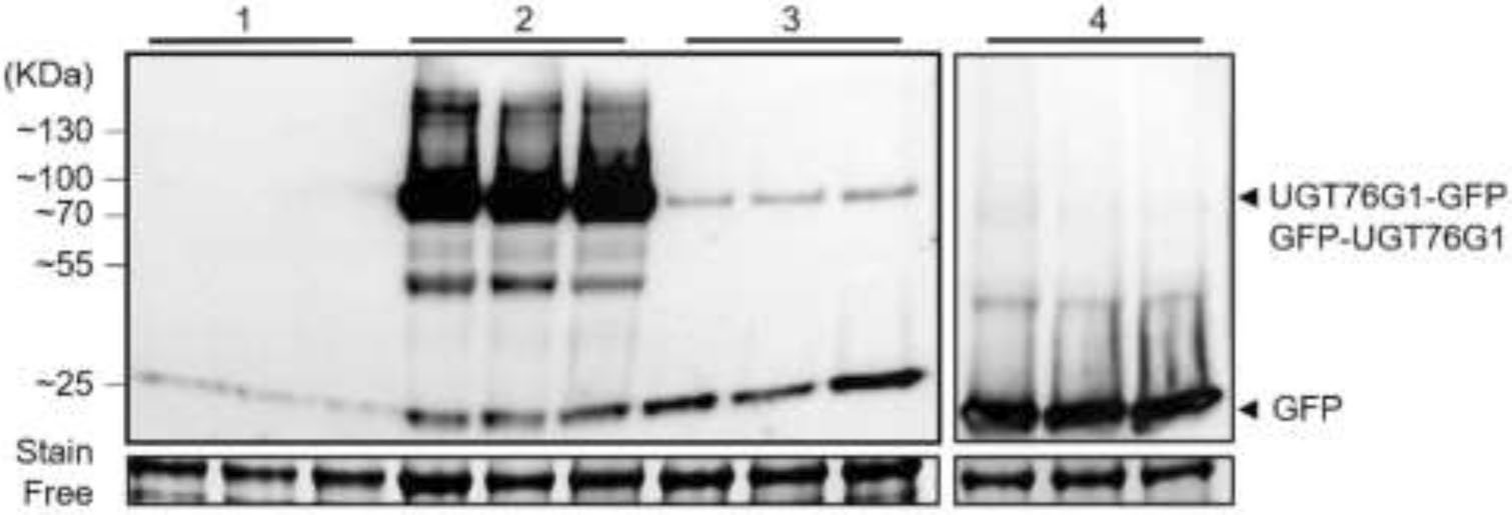
Anti-GFP western blot analysis of recombinant proteins expressed in *N. benthamiana* at 5 days post-infiltration (dpi). Recombinant proteins are shown with arrows. The figure presents 3 biological repetitions per condition. 1: non-transformed plants; 2: p35S::UGT76G1-GFP; 3: p35S::GFP-UGT76G1; 4: p35S::GFP.

To characterize UGT76G1, GFP was added in N or C terminus of the protein as tag may interfere with protein localization or activity^20,21^. Both constructs p35S::UGT76G1-GFP and p35S::GFP-UGT76G1 were agroinfiltrated in *N. benthamiana* leaves, using *A. tumefaciens*. At 5 days post-infiltration (dpi), specific bands are observed on western blot at around 77 kDa, the expected size of UGT76G1-GFP and GFP-UGT76G1 proteins (Fig. 1). Strong bands corresponding to UGT76G1-GFP confirm the accumulation of the protein while weak bands are detected for the N-terminally tagged version (see Supplementary Fig. S1 for longer acquisition signal). This indicates that although both versions of UGT76G1 recombinant proteins are expressed, it seems that GFP N-terminal tag may interfere with the accumulation of the protein. Bands of great intensity at around 25 kDa corresponds to expression of GFP as control.

### UGT recombinant proteins harbor a nucleo-cytoplasmic localization

**Figure 2:**
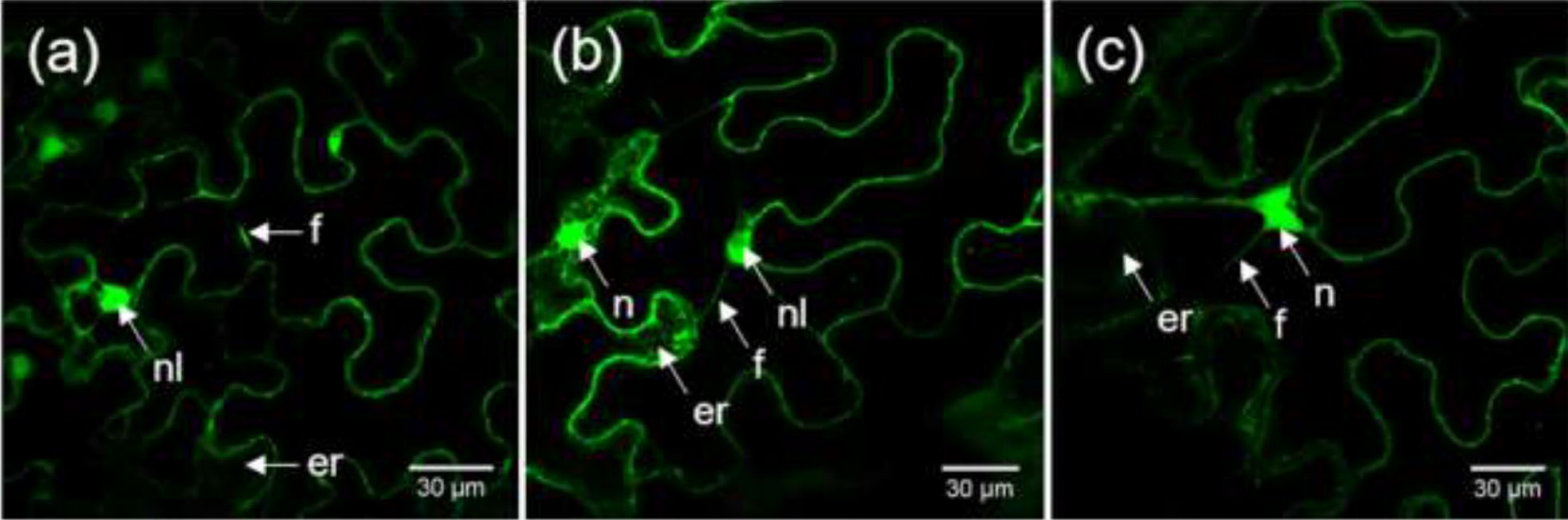
CLSM observations of recombinant proteins at 5 dpi (**a**) GFP, (**b**) GFP-UGT76G1, (**c**) UGT76G1-GFP. *er* endoplasmic reticulum, *f* cytosolic filaments, *n* nucleus, *nl* nucleolus.

Expression of recombinant proteins was verified by confocal laser scanning microscopy (CLSM) at 5 dpi. The GFP fluorescence was observed in leaves transformed with GFP, GFP-UGT76G1 and UGT76G1-GFP, confirming their expression (Fig. 2).

Recombinant GFP is visible in the cytosol for all three constructs due to the specific green fluorescence of filaments passing through the cell. Part of the specific fluorescence is observed in the endoplasmic reticulum, which appears as a diffuse green “grid” in the cell. This is probably due to the presence of recombinant proteins at the level of the maturation zone.

GFP fluorescence is also detected in the nucleus for all three constructs at 5 dpi (but not in the nucleolus). This localization could be due to passive diffusion of GFP. Some studies support the hypothesis of bidirectional diffusion of free GFP through nuclear pores, due to its low molecular weight^22,23^. GFP-UGT76G1 and UGT76G1-GFP being bigger, one hypothesis to explain their nuclear fluorescence could be the degradation of recombinant proteins allowing fragments of free GFP to diffuse into the nucleus. This hypothesis is supported by our western blot analyzes at 5 dpi, where specific GFP band (26.4 kDa) was revealed in the corresponding lanes (Fig. 1).

### *N. benthamiana* does not convert stevioside in steviol glycoside

**Figure 3:**
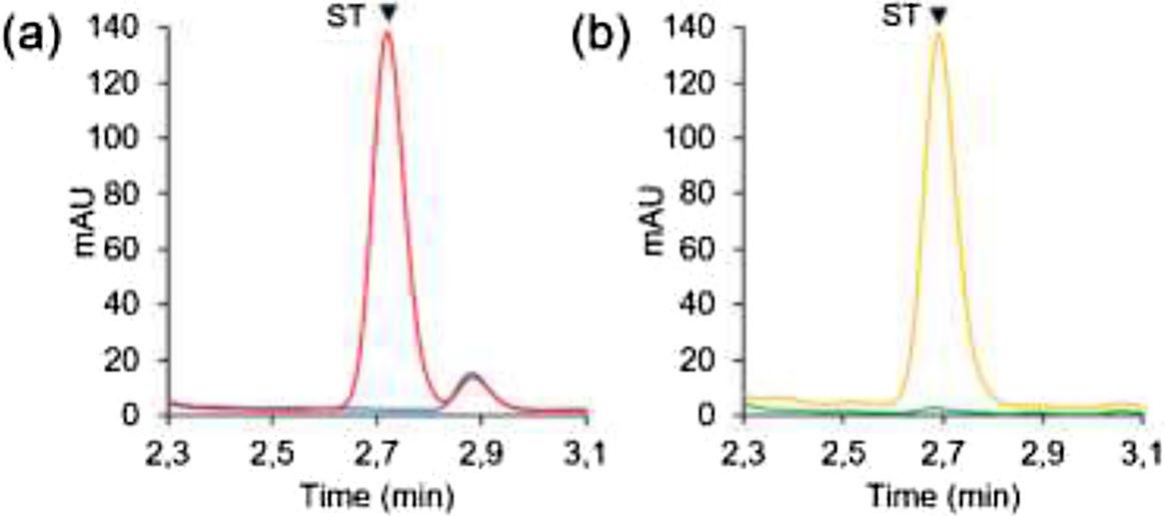
Glycosylation activity of non-transformed *N. benthamiana.* (**a**) In vitro test: a crude extract was produced from leaves of non-transformed *N. benthamiana*, and was analyzed by UPLC after a 2 h-incubation with (red) or without (blue) ST, (**b**) In planta test: leaves of non-transformed *N. benthamiana* were analyzed by UPLC, 24 h after infiltration of stevioside in leaves (yellow) or no infiltration (green). *ST* stevioside.

Stevioside (ST) and other SVglys were identified by their elution times and UV spectra using corresponding standards (see Supplementary Fig. S2). Chromatograms corresponding to leaves from non-transformed plants and that have been in presence of ST show a characteristic peak at around 2.7 min corresponding to ST (red and yellow curves), contrary to the negative control (blue and green curves) (Fig. 3). No new peak appears close to that of ST, meaning that non-transformed *N. benthamiana* does not have a UGT able to transform ST into Reb A. This result was confirmed by both functionality tests and allowed the continuation of activity trials on plants expressing UGT76G1.

### UGT76G1 expressed in *N. benthamiana* produces rebaudioside A from stevioside

**Figure 4:**
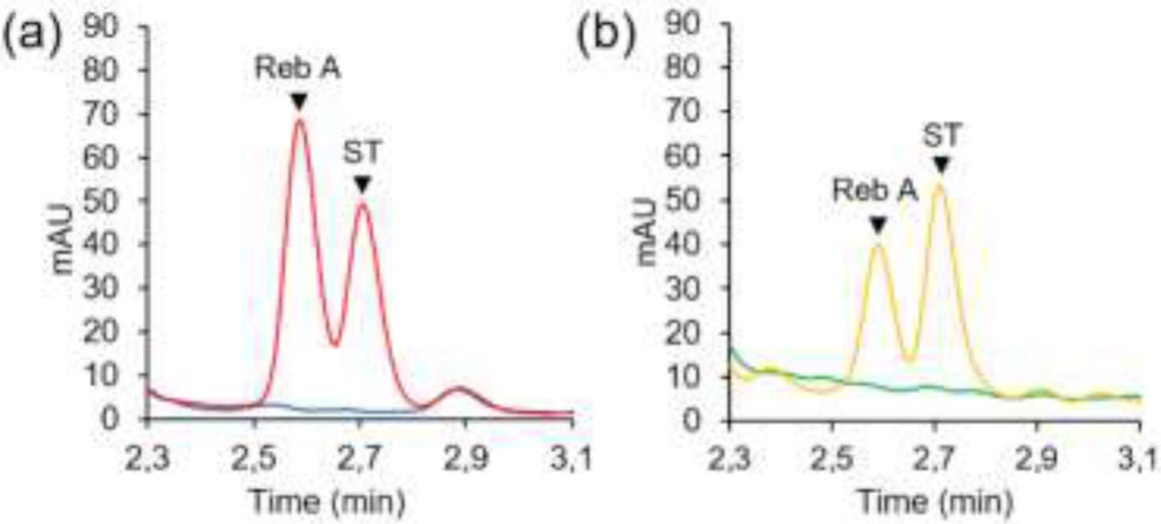
Activity of UGT76G1 expressed in *N. benthamiana* at 5 dpi. (**a**) In vitro test: a crude extract was produced from leaves of transformed *N. benthamiana* and was analyzed by UPLC after a 2 h-incubation with (red) or without (blue) ST, (**b**) In planta test: leaves of transformed *N. benthamiana* were analyzed by UPLC, 24 h after infiltration of ST in leaves (yellow) or no infiltration (green). *Reb A* rebaudioside A, *ST* stevioside.

In vitro and in planta activity tests were then conducted on *N. benthamiana* leaves expressing UGT76G1. According to the chromatograms, leaves expressing UGT76G1 and that have not been in the presence of ST (blue and green curves), cannot synthesize Reb A naturally as no peak specific to SVglys appears (Fig. 4). In the presence of ST (red and orange curves), the UGT76G1 expressed in *N. benthamiana* catalyzes β-1,3 glucosylation to produce Reb A, whose characteristic peak appears on the chromatograms at 2.6 min.

### GFP tag does not affect UGT76G1 functionality

**Figure 5:**
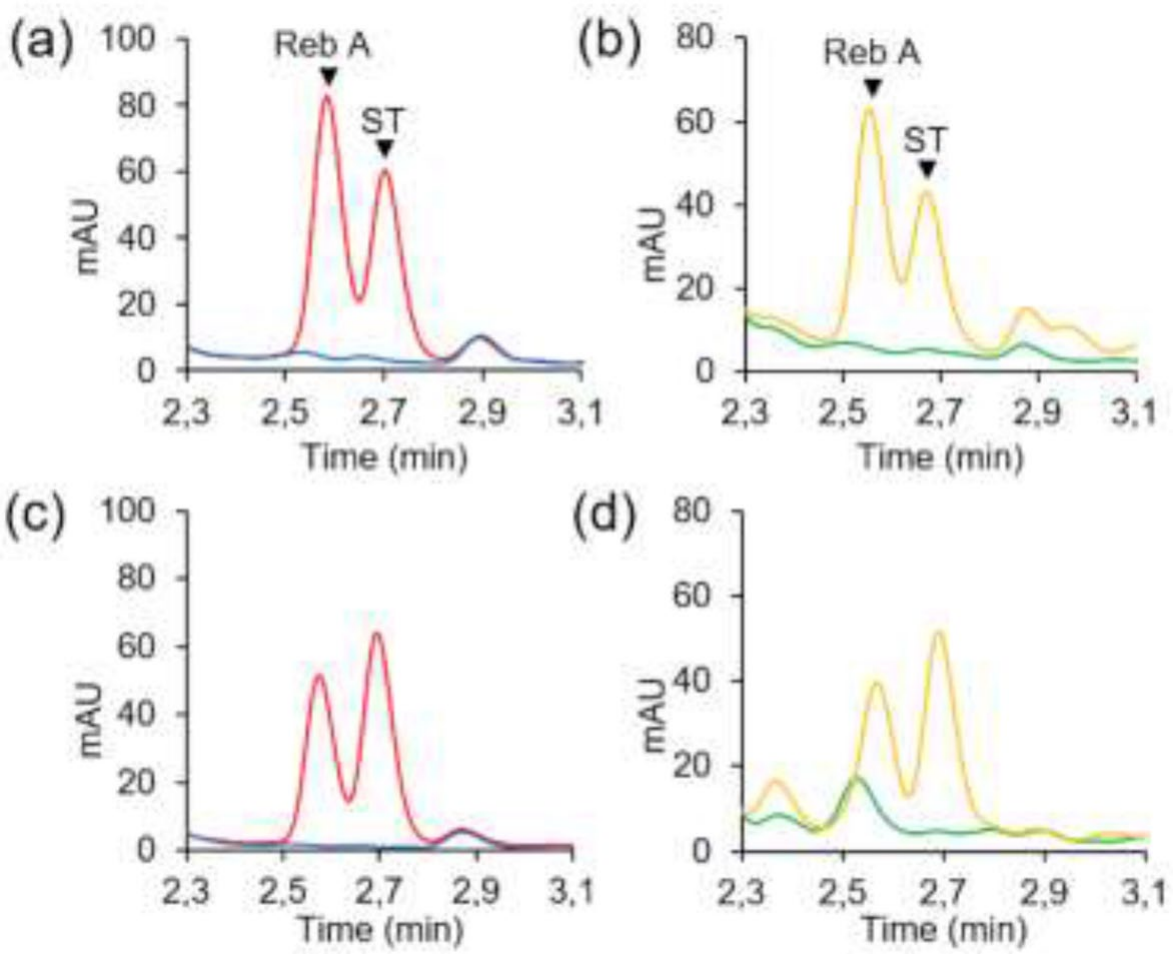
Activity of UGT76G1-GFP and GFP-UGT76G1 expressed in *N. benthamiana* at 5 dpi. (**a**, **c**) In vitro test, after a 2 h-incubation of a leaf extract with (red) or without (blue) ST, (**b**,**d**) In planta test, 24 h after infiltration of ST in leaves (yellow) or not (green). (**a**, **b**) p35S::UGT76G1-GFP, (**c**, **d**) p35S::GFP-UGT761. *Reb A* rebaudioside A, *ST* stevioside. Original chromatogram for (**a**) (red) is given in Supplementary Fig. S4 and compared with SVglys standards for peak identification.

Functionality of fusion proteins UGT76G1-GFP and GFP-UGT76G1 was then tested using the two methods. According to the chromatograms, the negative controls (corresponding to blue and green curves) do not synthetize Reb A as no specific peak appears (Fig. 5). In the presence of ST (red and orange curves), UGT76G1-GFP and GFP-UGT76G1 proteins expressed in *N. benthamiana* catalyze the β-1,3 glucosylation to produce Reb A. The same results have been observed for UGT76G1 coupled with a polyhistidine tag UGT76G1-6xHis and 6xHis-UGT76G1 (see Supplementary Fig. S3).

These two methods of functional characterization can therefore be used for UGTs that intended to be purified or to be localized at the cellular level, provided that the conformation of the active site is conserved, and that it is accessible despite the GFP or 6xHis tags.

### UGT76G1 produces rebaudioside C and F from steviol glycosides subtrates

An in vitro activity test was carried out from a crude extract of *N. benthamiana* leaves expressing UGT76G1 and put in the presence of the Genotype E’s extract obtained from dried leaves. The objective was to evaluate the activity of the enzyme in the presence of several substrates. Genotype E used to produce the SVglys extract mainly accumulates ST and Dulc A, due to the non-functionality of the β-1,3 glucosylation, as previously shown^10^.

**Figure 6:**
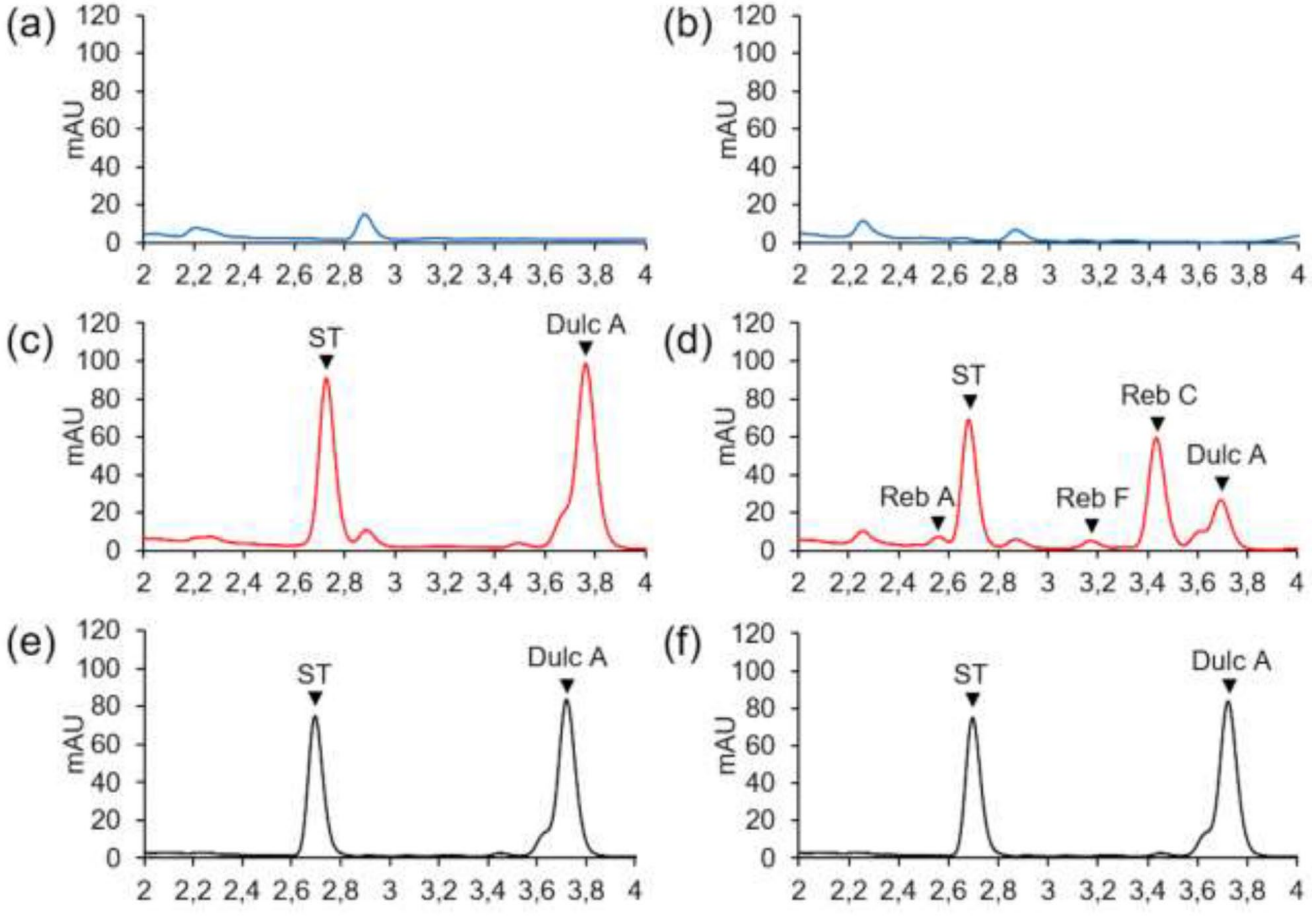
Functionality of UGT76G1 in presence of several SVglys subtrates: in vitro activity of *N. benthamiana* crude extracts incubated without (**a**,**b**) or with (**c**,**d**) SVglys extract from Genotype E. (**a**,**c**) Non-transformed *N. benthamiana*, (**b**,**d**) *N. benthamiana* expressing UGT76G1 at 5 dpi, (**e**,**f)** SVglys extract from Genotype E. *Dulc A* dulcoside A, *ST* stevioside, *Reb A* rebaudioside A. Original chromatogram for (**e**,**f**) is given in Supplementary Fig. S4 and compared with SVglys standards for peak identification. Original chromatograms for (**d**–**f**) are given in Supplementary Fig. S5 for comparison of SVglys presence/absence.

Chromatograms corresponding to the negative controls show that (1) the non-transformed *N. benthamiana* leaves do not naturally produce SVglys (Fig. 6a), (2) they do not catalyze the β-1,3 glucosylation of the SVglys of the Genotype E’s extract (Fig. 6c) (3) and that leaves of *N. benthamiana* expressing UGT76G1 do not produce SVglys (Fig. 6b).

Chromatograms relative to *N. benthamiana* leaves expressing UGT76G1 and put in presence of the Genotype E’s extract show additional peaks with respect to ST and Dulc A (Fig. 6d). These molecules have retention times corresponding to Reb A, C and F. The surface area of the Dulc A peak decreased compared to that of the SVglys extract alone (Fig. 6e,f), in favor of an increase in the area of the Reb C peak, probably corresponding to glucosylation of Dulc A. Concerning Reb F, its precursor is not identified among SVglys peaks appearing on the chromatograms. The outgoing peak near the Dulc A seems to decrease compared to the SVglys extract alone, so it could correspond to the Reb F precursor.

Considering the proportions of Reb A and Reb C relative to those of their respective precursors, only 7.8 ± 1.3% (n = 3) of ST are converted into Reb A on average against 73.5 ± 4.9% of Dulc A converted in Reb C (n = 3), after 2 h. This isoform seems to be preferentially active with Dulc A rather than with ST. If it was the case, is could be due to easier access to the β-1,3 glycosylation site on Dulc A compared to ST. Indeed, these two compounds differ only in the nature of the β-1,2 sugar: Dulc A has a rhamnose and ST, a glucose. This hypothesis could be verified with affinity measurements on a purified protein.

## Discussion

In this work, two types of activity tests were used to evaluate the functionality of a plant UGT, directly from a crude extract or plants expressing the protein of interest. These tests are based on an on/off detection system of the enzyme products and are not intended for quantification. These methods showed that recombinant UGT76G1 catalyzes the conversion of ST to Reb A, as expected^14^. The tests also revealed that the β-1,3 glucosylation activity of UGT76G1 was not affected by the addition of a GFP or polyhistidine tag (6xHis), both in C- and N-terminal. This allows with a single vector, to test both the activity of the enzyme and research its localization in the cell, or to purify it for a more refined enzymatic characterization.

Through expression in *N. benthamiana*, UGT76G1 was localized in cytosol, as previously shown^24^. This compartmentalization is logical considering the role of glycosylation of UGT76G1, which occurs after synthesis of the aglycone in the chloroplast and the endoplasmic reticulum and before the storage of glycosides in the vacuole. The GFP fusions were also detected in ER which may be explained by presence of the recombinant UGT76G1 in the maturation area. Fluorescence of UGT76G1-GFP and GFP-UGT76G1 were also observed in nucleus. The same nuclear fluorescence pattern was already observed for some plant UGTs as GFP-tagged UGT85A1 from *Arabidopsis*^25^ or GFP tagged *Ta*UGT3 of *Triticum aestivum* expressed in onion epidermis cells^26^. A nuclear localization was also obtained for the stevia’s UGT74G1 expressed in onion epidermal cells^24^, for which the authors advocated for a weak diffusion of the fusion protein into the nucleus. Even if passive diffusion decreases with protein size, it was shown that proteins larger than 60 kDa passively diffuse from cytosol into the nucleus, thus accumulating over time^27,28^. The authors found that passive diffusion does not have a molecular mass threshold, contradicting the view that the maximal size for protein diffusion through the nuclear pore is around 60 kDa^27,28^. Another alternative to explain stevia’s UGTs localization in nucleus supports the presence of a nuclear addressing and export signal although it was never shown^24^. However, we cannot exclude the presence of the functional protein in the nucleus, as it was shown in *Zea mays* root cells for the *trans*-zeatin *O*-glycosyltransferase enzyme, for which nuclear localization was confirmed by immunodetection^29,30^. In our case, part of the GFP fluorescence in the nucleus may also come from fusions degradation.

Many chemical phenotypes have been observed in our *S. rebaudiana* collection^8^. The diversity of SVglys profiles thus makes it possible to produce numerous extracts directly obtained from the plant, with different combinations of substrates. For example, Genotype E is not able to produce Reb A and Reb C^10^. Extraction of SVglys from Genotype E’s leaves allow production of an extract rich in ST and Dulc A, which was used as substrates for UGT76G1. The method developed in vitro showed that UGT76G1 contained in a crude extract of *N. benthamiana* catalyzes the β-1,3 glucosylation of Dulc A to form Reb C, from Genotype E’s extract. The crude extract containing UGT76G1 also produces Reb F from the *S. rebaudiana* extract, confirming the assumption previously made^8^, although the precursor of this SVgly was not identified on the chromatograms. Usually, methods to test activity in vitro require purification of the recombinant protein. Here, the developed method allows to test the functionality of several UGTs simply from a crude extract, using as substrates individual SVglys as well as SVglys synergies, moving closer to in vivo conditions. With identical crude extract/substrate ratio and reaction time, relative substrate affinity of a UGT could be investigate, by testing several individual and combined substrates. It could also be adapted to test UGTs thought to be involved in minor or higher glycosylations of SVglys, requiring a finer detection system such as mass spectrometry.

The in planta method is unique in that it infiltrates the substrate directly into the *N. benthamiana* leaf expressing the protein of interest. This method has previously been used but remains rather unusual in protein functionality studies and has never been used to study *S. rebaudiana* UGTs. For example, *Pg*UGT5 and *Pg*UGT5b from *Picea glauca* involved in pungenol glycosylation were expressed in *N. benthamiana*, and their substrate was directly injected into the transformed leaves^31^. However, *N. benthamiana* exhibited endogenous glycosylation activity leading to a basal production of the detected products. The use of this type of method appears to be appropriate for the study of UGTs involved in the biosynthesis of SVglys as it may limit this endogenous phenomenon. Indeed, the SVglys pathway does not exist in *N. benthamiana*, which restricts the existence of UGTs that can be recruited for the glycosylation of SVglys.

Taken together, these methods are effective tools to screen functionality of recombinant UGTs suspected to be involved in SVglys biosynthesis. Once expression vectors are produced and *N. benthamiana* is transformed, the user can choose the best method. The in vitro method only requires one expression vector and is suited to quickly screen several substrates and non-characterized UGTs. The in planta method uses binary vectors from pGWBs series and allows simultaneously functional screening close to the in vivo conditions, and several applications for further analysis, such as subcellular localization or purification.

## Material and methods

### Biological material

#### Nicotiana benthamiana

*Nicotiana benthamiana* seeds used for transient transformation were provided by LRSV (Toulouse, France). Each seed was sown in a 7 × 7 cm plastic pot in a special seedling and cuttings potting soil. The seeds were sprouted in moisture-saturated plastic mini-greenhouses (closed aeration) to allow germination and good plant development. The seedlings were placed under long day conditions: 16 h of day and 8 h of night, at a temperature of 20–23 °C. After the appearance of the first 3 leaves, the vents of the mini-greenhouses were opened (weekly watering) then the lids of the mini-greenhouses removed (daily watering), in order to accustom the plants to the ambient hygrometry.

#### Stevia rebaudiana

Our germplasm is derived from selection cycles of Criolla and Morita populations. Genotype E previously studied, has a non-functional UGT76G1^10^ and doesn’t produce Reb A and Reb C, and accumulates high proportions of Dulc A and ST. Genotype E was cultivated in 1-L pot in greenhouse under long days (16 h of daylight), to maintain plants in a vegetative stage and favor biomass production.

### Production of expression vectors

The coding sequence of genotype A’s UGT76G1 of our previous study was used for the experiments^10^. Primers used for coding sequence amplification of UGT76G1 were used to design attB specific primers, for native expression and C- and N-term fusions (Table 1).

**Table 1:** UTG76G1 specific primers used to synthetize attB-PCR products.

Orientation	Primer	Usage
Forward	AAAAAGCAGGCTATGGAAAATAAAACGGAGACCA	Native expression
		C-term fusion
	AAAAAGCAGGCTTCATGGAAAATAAAACGGAGACCA	N-term fusion
Reverse	AGAAAGCTGGGTTTACAACGATGAAATGTAAGAAACTAG	Native expression
		N-term fusion
	AGAAAGCTGGGTGCAACGATGAAATGTAAGAAACTAG	C-term fusion

The synthesis of attB-UGT76G1 PCR products was performed in two steps. Platinum Taq DNA Polymerase High Fidelity (Thermo Fisher Scientific) was used for the two PCR, according to manufacturer recommendations. The first PCR (50 µL) consisted in amplifying purified clones containing UGT76G1 of the Genotype A with the specific primers of Table 1. The cycling parameters described in Table 2 were used.

**Table 2:** Cycling parameters for the first step PCR.

Denaturation	2 min	95 °C	
Denaturation	15 s	94 °C	10 cycles
Annealing	30 s	53.3 °C	
Elongation	1 min/kb	68 °C	

Ten microliters of the first PCR reaction were transferred to a 40 µL PCR mixture containing 40 pmol each of the attB1 and attB2 adapter primers (AttB1: GGGGACAAGTTTGTACAAAAAAGCAGGCT, AttB2: GGGG ACCACTTTGTACAAGAAAGCTGGGT.). The second PCR (50 µL) used the cycling parameters described in Table 3.

**Table 3:** Cycling parameters for the second step PCR.

Denaturation	2 min	95 °C	
Denaturation	15 s	94 °C	5 cycles
Annealing	30 s	45 °C	
Elongation	1 min/kb	68 °C	
Denaturation	15 s	94 °C	15–20 cycles
Annealing	30 s	55 °C	
Elongation	1 min/kb	68 °C	
Elongation	10 min	68 °C	

Entry clones were created by BP recombination between the attB-UGT76G1 purified products and the vector pDONR/Zeo (Invitrogen, Thermo Fisher Scientific) with the BP Clonase enzyme (Invitrogen, Thermo Fisher Scientific), following the manufacturer recommendations. Library Efficiency DH5α (Thermo Fisher Scientific) competent cells were transformed by heat shock at 42 °C during 45 s with the entry clones. Positive clones were selected and sequenced to confirm the insertion of the transgene and the correct reading frame for fusion proteins. Entry clones were then purified with the Wizard Plus Minipreps DNA Purification System (Promega) according to the manufacturer recommendations. Expression clones were produced by a LR recombination between the entry clones and the binary vectors pGWBs^32^, with the LR Clonase enzyme (Invitrogen, Thermo Fisher Scientific). The vectors pGWBs were provided by T. Nakagawa (Shimane University, Japan). Library Efficiency DH5α (Thermo Fisher Scientific) competent cells were transformed by heat shock at 42 °C during 45 s with the expression clones. Positive clones were selected and purified with the purification system NucleoBond Xtra Midi (Macherey–Nagel) following the manufacturer recommendation.

### *Agrobacterium tumefaciens* transformation

The LBA4404 strain was used for transformation (ElectroMAX *A. tumefaciens* LBA4404 Cells, Invitrogen, Thermo Fisher Scientific). Cells were transformed with expression vectors in electroporation cuvettes, during 5 ms at 1.8 kV, 200Ω, 25 µF. After electroporation, 800 µL of YEB medium were added and cultures were incubated at 28 °C during 2 h at 180 rpm. Cells were spread on LB plates with rifampicin, streptomycin, and hygromycin (at 100 µg/mL final) and incubated during 48 h at 28 °C. Positive clones were selected and pricked out in LB liquid medium.

### *Nicotiana benthamiana* transformation

For each construct, a 200 mL-culture with appropriate antibiotics was prepared starting from 40 µL of a glycerol stock. Cells were grown at 28 °C, 150 rpm until 1.8 < DO_600nm_ < 2.0. They were then centrifugated 20 min at 1,000*g*. Media was removed and cells were washed with the resuspension solution (10 mM MgCl_2_, 10 mM MES-K pH 5.6). After wash, pellets were resuspended in the resuspension solution containing acetosyringone 100 µM. An aliquot of the bacterial solution was sampled and diluted in the resuspension solution with acetosyringone qsp. the desired volume until 0.7 < DO_600nm_ < 0.9. Cells were incubated during 2 h at ambient temperature. *Agrobacterium* was infiltrated into *N. benthamiana* leaves with a 5 mL-syringe applied on the lower face. As control of plant transformation, *A. tumefaciens* LBA4404 strain containing a vector pEAQ-HT-Dest1-GFP was used^33^. This strain was provided by L. Hoffman (University of Toulouse, France). Vectors containing *UGT76G1* and *GFP* were co-infiltrated with the co-suppressor p19 (with a bacterial solution volume ratio 2:1) as it enhances recombinant protein expression (see Supplementary Fig. S6). The vector pCB301-p19 was provided in a LBA4404 strain by J. Win (Sainsbury Laboratory, United-Kingdom).

### Protein analysis

#### Sample preparation

Leaves were sampled 5 days post infiltration. Immediately after sampling, leaves were frozen in liquid nitrogen. They were ground into a fine powder with a ball mill MM300 (Retsch) and stainless-steel balls (2 mm Ø). One volume of sample was extracted in 2 volumes of extraction buffer (Triton X100 0.25%, DTT 10 mM, Halt Protease Inhibitor Cocktail [Thermo Fisher Scientific) 1X, qsp. GTEN buffer (glycerol 10%, Tris–HCl pH 7.5 150 mM, EDTA 1 mM, NaCl 150 mM)]. Samples were incubated 10 min at 4 °C under gentle stirring. After centrifugation 10 min at 4 °C, 3,000*g*, supernatants were sampled and conserved for protein analysis. Proteins were quantified using the Bradford method. For western blot, 20 µg of proteins were denatured 5 min at 96 °C using 5 volumes of protein extract and 1 volume of Laemmli 6X. Samples were centrifugated 30 s at 13,000*g* and supernatants were used for electrophoresis.

#### Western blot

Proteins were loaded on a 1 mm gel prepared with TGX Stain-Free FastCast Acrylamide Kit, 10% (BioRad) according to manufacturer specifications. Proteins were separated in a Mini-Protean Tetra Cell System (BioRad) at 250 V (400 mA) during 30 min. Separated proteins were transferred on nitrocellulose membrane with a Trans-Blot Turbo Transfer System (BioRad) at 25 V during 3 min per gel. Membranes were saturated during 1 h under strong stirring with 5% fat free milk dissolved in TBS Tween 0.1%. Then, they were incubated with primary antibodies prepared at 1/1,000 in BSA 1X TBS-Tween 1X during 2 h at ambient temperature. For blotting of GFP-tagged proteins, monoclonal anti-GFP antibodies produced in mouse were used respectively (Sigma). After incubation with primary antibodies, membranes were washed three times with TBS-Tween 1X during 10 min and incubated under stirring during 1 h with secondary antibodies prepared at 1/6,000 in BSA 1X TBS Tween 1X. Secondary antibodies anti-mouse IgG (H + L)-HRP conjugated antibodies produced in goat were used (BioRad). Membranes were washed three times with TBS-Tween 1X during 10 min. Specific bands were revealed with Clarity Western ECL Substrate (BioRad) and membranes were observed with a ChemiDoc XRS + system (BioRad). For the revelation step, anti-GFP blots were exposed for 60s except for GFP controls (3s) to avoid overexposition. The 60s exposition for the GFP control is shown on Supp fig 1.

#### Microscopy

Microscopy observations were realized at the platform FR AIB (Toulouse, France) at 5 days post-infiltration. GFP fluorescence (λ_excitation_ = 488 nm; λ_emission_ = 510 nm) was observed with a confocal laser scanning microscope (CLSM) TCS SP8 (Leica) and a HC Fluotar L25X/0.95 W VISIR (Leica) lens. Pictures were taken with the software LAS (Leica) and were processed with the software ImageJ (1.52a).

### Activity of recombinant proteins

#### Production of the SVglys extract

Genotype E’s leaves (complete stems) were sampled and dried at 50 °C. Then, they were ground with a mortar and pestle. SVglys were extracted from 10 g of Genotype E’s dry leaves during 30 min at 50 °C under stirring in 200 mL of ultrapure water. The extract was filtrated with absorbent paper to remove leaf powder and fragments.

#### In planta: infiltration of substrate in transformed leaves

At 5 dpi, transformed leaves were infiltrated by syringe with stevioside 5 mM. After 24 h, leaves were sampled separately and dried 48 h at 50 °C. Then, they were grinded with piston pellet Eppendorf and steviol glycosides were extracted 30 min at 50 °C in 200 µL of ultrapure water. Extracts were filtrated at 0.2 µM and analyzed by UPLC.

#### In vitro: enzymatic assay on crude extract expressing recombinant protein

For each construct, two transformed leaves were sampled at 5 dpi and immediately ground in ice with 500 µL of cold reaction buffer (Tris–HCl 100 mM, pH 8.0; MgCl_2_ 3 mM; BSA 10 µg mL^−1^). Extracts were centrifugated 2 min at 2,000*g* at 4 °C, and 180 µL of supernatant were transferred in two new tubes. Twenty microliters of reaction buffer were added in one of the tubes to constitute the negative control. Twenty microliters of substrate were added in the other tube. The substrates were alternatively stevioside 5 mM or SVglys extract for the Genotype E, mainly composed of ST and Dulc A.

Tubes were homogenized by gentle flipping. After 2 h at 30 °C, reactions were stopped by addition of 10 µL TFA 20%. Samples were centrifugated 5 min at 2,000*g*. Supernatants were filtrated at 0.2 µm and analyzed by UPLC.

#### UPLC analysis of steviol glycosides

Liquid chromatography was performed under isocratic conditions on a Dionex UltiMate 3,000 system from ThermoScientific consisting of a DGP-3600RS pump, a WPS-3000 TRS autosampler, a TCC-3000 SD column compartment maintained at 40 °C and a DAD-3000 diode array detector set to a wavelength of 195 nm. The flow rate and sample injection volume were 0.3 mL min^−1^ and 1 μL, respectively. Steviol glycosides were separated with a reverse phase (RP) column Kinetex C18 (150 × 2.1 mm, 1.7 μm) (Phenomenex, USA), maintained at 40 °C. Solvents used as mobile phase were 32% acetonitrile and 68% ultra-high-purity water acidified with 0,1% formic acid (pH 2.6). A mix of 9 SVglys standards (JECFA mixture) was purchased from Chromadex, Inc. (Irvine, CA, USA) and were used to identify ST, Dulc A, Reb A and C. Chromatogram of standards and UV spectrum of ST are given in the Supplementary Fig. S2.

## Supplementary information

Supplementary Figures.
