# Supplementary material for: Development of screening methods for functional characterization of UGTs from Stevia rebaudiana
Source: Sci Rep. 2020 Sep 15;10:15137. doi: 10.1038/s41598-020-71746-9 (PMC7493886; doi:10.1038/s41598-020-71746-9)
Supplement: Supplementary file 1 — Supplementary Figures. [file 41598_2020_71746_MOESM1_ESM.pdf]

Supplementary Information

Development of screening methods for functional characterization of UGTs from *Stevia rebaudiana*

Eva Petit, Monique Berger, Laurent Camborde, Veronica Vallejo, Jean Daydé, and Alban Jacques

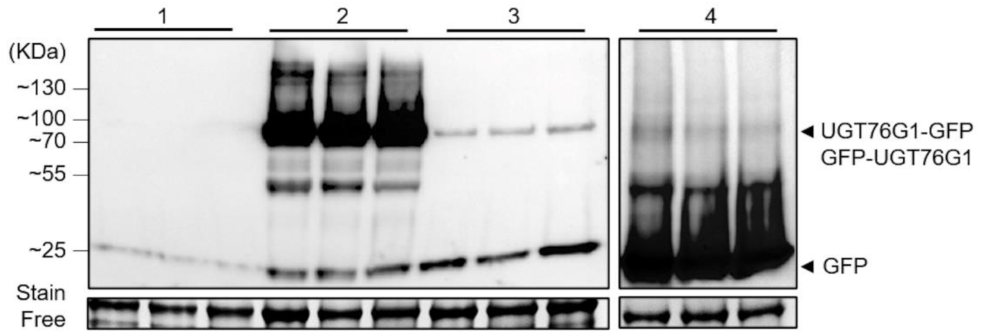

Figure S1. Anti-GFP western blot analysis of recombinant proteins expressed in *N. benthamiana* leaves after longer revelation step. Recombinant proteins are shown with arrows. The figure presents 3 biological repetitions per condition. 1: non-transformed plants; 2: p35S::UGT76G1-GFP; 3: p35S::GFP-UGT76G1; 4: p35S::GFP.

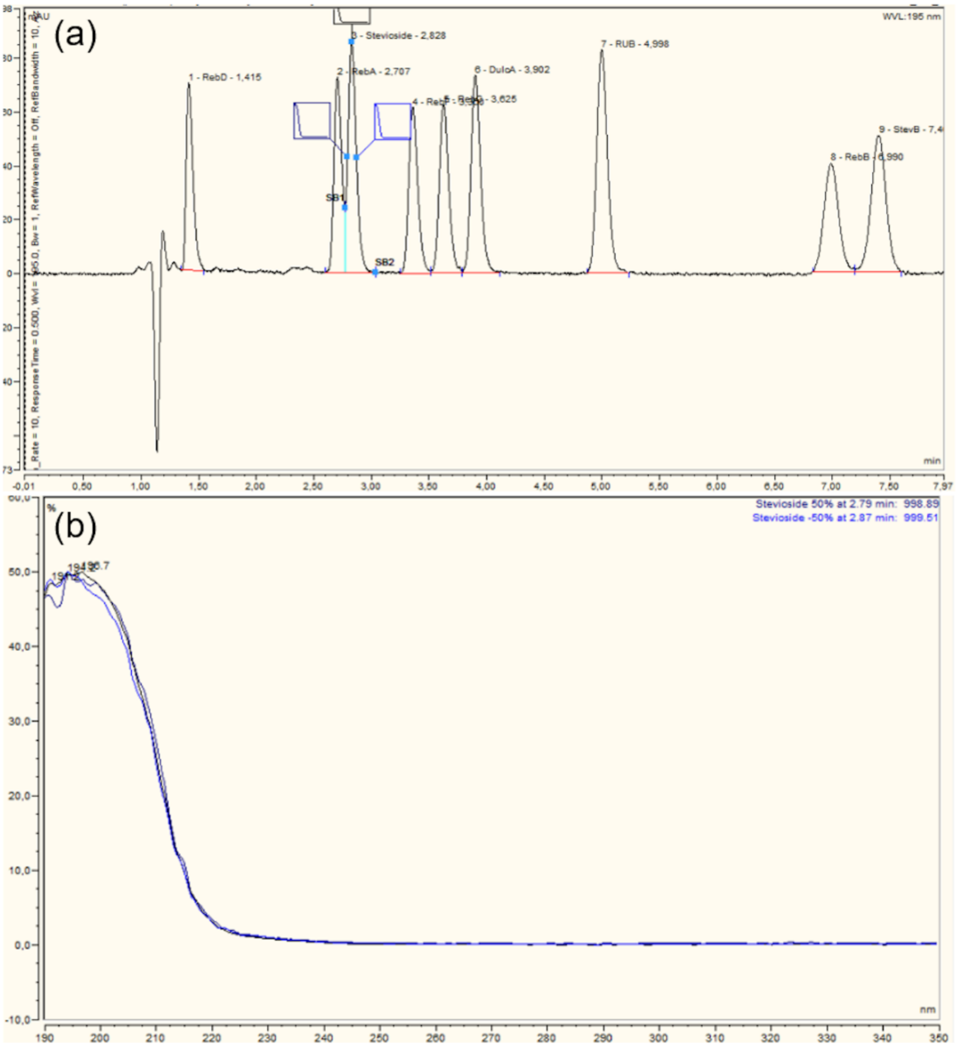

Figure S2. RP-UPLC separation and identification of SVglys standards (a) Chromatogram of the 9 SVglys standards (JECFA mixture) with the following order of elution: rebaudioside D, rebaudioside A, stevioside, rebaudioside F, rebaudioside C, dulcoside A, rubusoside, rebaudioside B, stéviolbioside; (b) UV spectrum of stevioside at 195 nm obtained by diode array detection (all the SVglys have the same spectrum as steviol, which is the only chromophore part of the molecule).

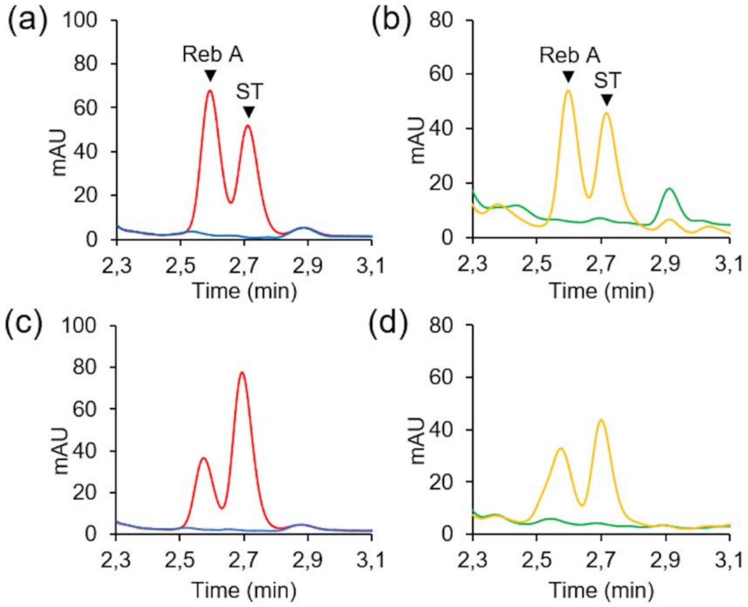

Figure S3. Activity of UGT76G1-6xHis and 6xHis-UGT76G1 expressed in *N. benthamiana* leaves. (a, c) *In vitro* test, after a 2h-incubation of a leaf extract with (red) or without (blue) ST; (b, d) *In planta* test, 24h after infiltration of ST in leaves (yellow) or not (green). a, b: p35S::UGT76G1-6xHis ; c, d p35S::6xHis-UGT761. Reb A: rebaudioside A; ST: stevioside.

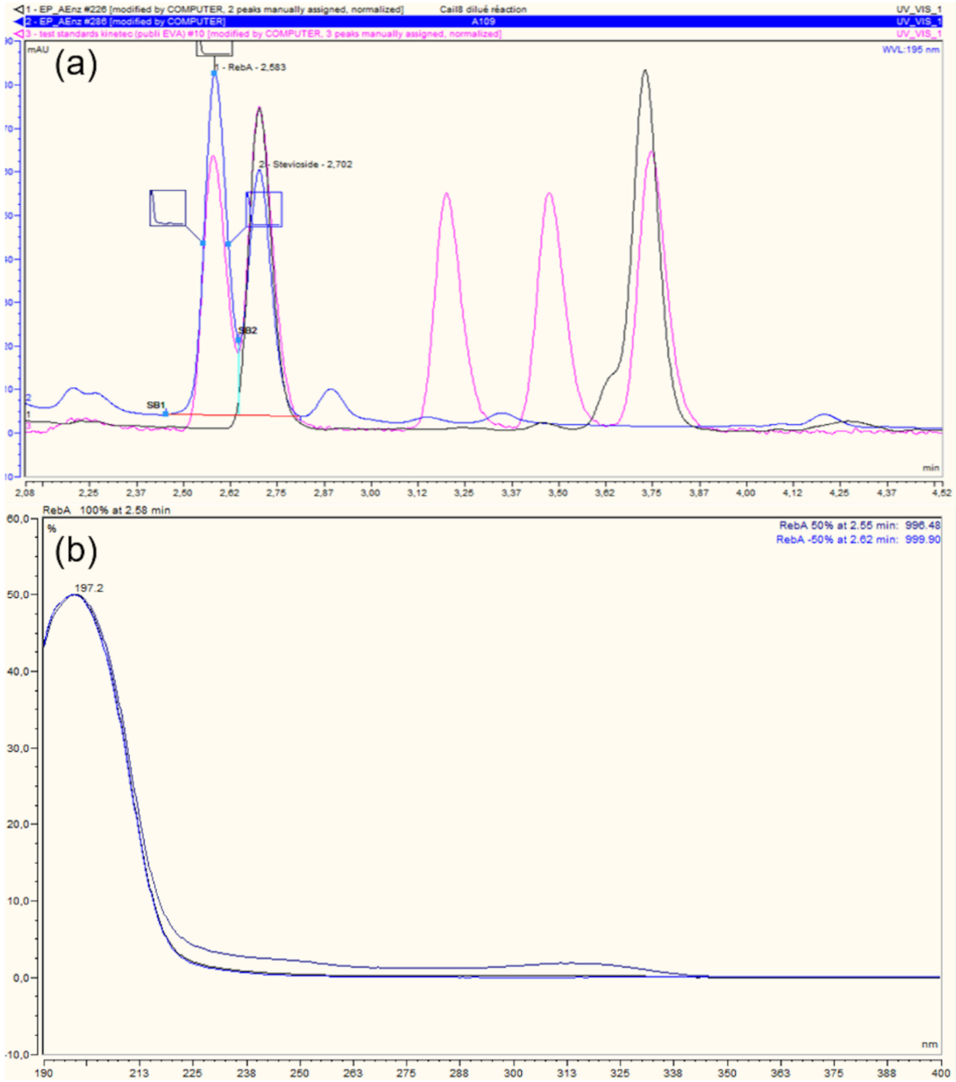

Figure S4. Peak identification using SVglys standards and UV spectra (a) Time spectra overlay. Pink line: JECFA standards with the following order of elution: rebaudioside A, stevioside, rebaudioside F, rebaudioside C, dulcoside A. Black line: SVglys extract from genotype E (corresponding to Fig. 6EF). Blue line: *N. benthamiana* extract expressing UGT76G1 incubated with stevioside (corresponding to Fig. 5A); (b) UV spectrum of rebaudioside A at 195 nm.

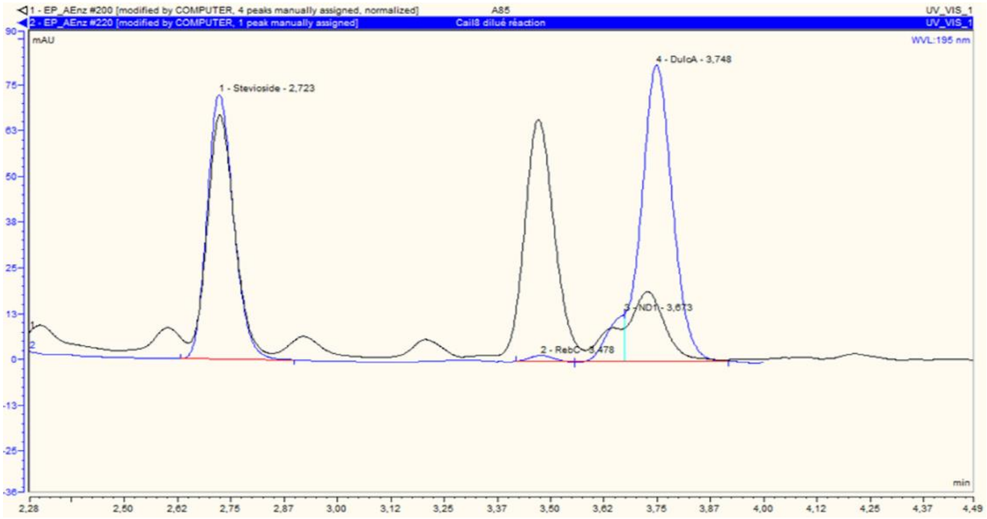

Figure S5. Time spectra overlay: comparison of the SVglys extract (from the genotype E) metabolized by a leaf extract of *N. benthamiana* expressing UGT76G1 (black line, corresponding to Fig. 6EF) or not metabolized (blue line, corresponding to Fig. 6D).

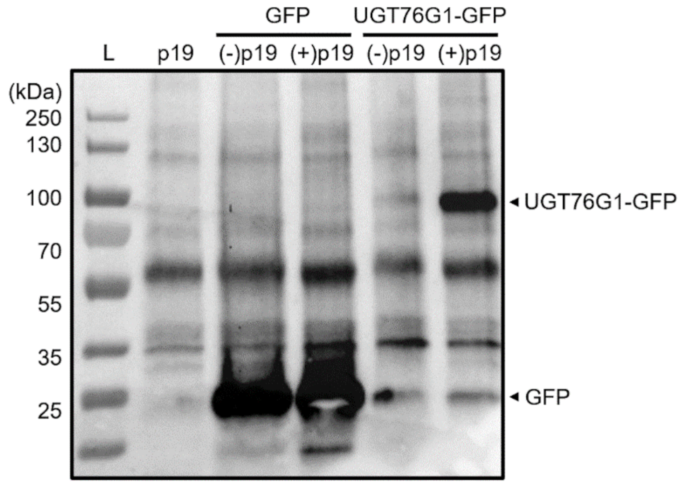

Figure S6. Anti-GFP western blot analysis of recombinant proteins expressed in *N. benthamiana* leaves. Here, UGT76G1-GFP was expressed in *A. tumefaciens* strain GV3101 provided by LRSV (Toulouse, France). Proteins were measured and normalized using a method of Bradford (BioRad Protein Assay). Arrows show recombinant proteins. p19: plants were only transformed with p19 co-suppressor; (+)p19: plants were transformed with a bacterial volumetric ratio 2:1 (GFP:p19 or UGT76G1-GFP:p19); (-)p19: plants were transformed with a bacterial volumetric ratio 2:1 (resuspension solution:p19); L: ladder (kDa).
